# Complete Genome Sequence of Polypropylene Glycol- and Polyethylene Glycol-Degrading *Sphingopyxis macrogoltabida* Strain EY-1

**DOI:** 10.1128/genomeA.01399-15

**Published:** 2015-12-03

**Authors:** Yoshiyuki Ohtsubo, Yuji Nagata, Mitsuru Numata, Kieko Tsuchikane, Akira Hosoyama, Atsushi Yamazoe, Masataka Tsuda, Nobuyuki Fujita, Fusako Kawai

**Affiliations:** a Department of Environmental Life Sciences, Graduate School of Life Sciences, Tohoku University, Sendai, Japan; b Biological Resource Center, National Institute of Technology and Evaluation, Tokyo, Japan; c Center for Fiber and Textile Science, Kyoto Institute of Technology, Kyoto, Japan

## Abstract

Strain EY-1 was isolated from a microbial consortium growing on a random polymer of ethylene oxide and propylene oxide. Strain EY-1 grew on polyethylene glycol and polypropylene glycol and identified as *Sphingopyxis macrogoltabida*. Here, we report the complete genome sequence of *Sphingopyxis macrogoltabida* EY-1. The genome of strain EY-1 is comprised of a 4.76-Mb circular chromosome, and five plasmids. The whole finishing was conducted *in silico*, with aids of computational tools GenoFinisher and AceFileViewer. Strain EY-1 is available from Biological Resource Center, National Institute of Technology and Evaluation (Tokyo, Japan) (NITE).

## GENOME ANNOUNCEMENT

*Sphingopyxis macrogoltabida* EY-1, a Gram-negative polyethylene glycol (PEG)- and polypropylene glycol (PPG)-utilizing strain, was isolated and identified (1), based on 16S rDNA analysis (AB255383) and the proposal by Takeuchi et al. (2). This strain was deposited to Biological Resource Center, National Institute of Technology and Evaluation (Tokyo, Japan) (NITE). The strain possessed a PEG dehydrogenase gene (AB255378) homologous to that from *Sphingopyxis macrogoltabida* 103 (3) and showed PPG dehydrogenase activity.

The strain EY-1 genome was sequenced using 454 GS-FLX Titanium (Roche) and HiSeq and MiSeq systems (Illumina). A fragment library was constructed for the 454 GS-FLX sequencing, and mate-pair and pair-end libraries were constructed for Illumina sequencing. The mate-pair library was sequenced by MiSeq for 301 bp apiece from both ends to obtain 2.5-M pairs (5.0 M reads). Each pair of reads was processed by ShortReadManager (4) (SRM) to categorize it into three classes of long-distance mate-pairs (MP), short-distance pair-end (PE), and single-end (SE), while read sequences were trimmed for adaptor and inverted as needed, to make the pair inward-facing. The reads were subjected to SRM trimming, in which 21-mers appearing only once was regarded as sequence errors or corresponds to adaptor junctions. GS-FLX reads and MiSeq reads were used for assemble by Newbler version 3.0, in which 79,800 FLX reads (56 Mb), 0.96 M MP reads (148 Mb), 1.3 M PE reads (274 Mb), and 1.1 M SE reads (185 Mb) were used (662 Mb in total), and we obtained 17 scaffolds and 128 contigs. The finishing was conducted by using GenoFinisher (GF) and AceFileViewer (AFV) (4). The 51 repeat-induced gaps were closed by GF and AFV. For closing 21 gaps that were arisen by lack of reads (MiSeq reads were scarcely obtained from some GC-rich regions), an Illumina pair end library constructed by using a PCR-free kit was sequenced by HiSeq for 100 bp apiece from both ends to obtain 3.0-M pairs. HiSeq reads were assembled by Newbler and contigs thus obtained were searched for DNA sequences that fill the gaps. The complete sequence of the strain EY-1 genome comprised one circular chromosome of 4,757,879 bp, and five plasmids of sizes 196,952 bp, 58,892 bp, 34,474 bp, 30,496 bp, and 19,860 bp.

The finished sequence was confirmed by FinishChecker tool of GF, and then annotated by the NCBI Prokaryotic Genomes Automatic Annotation Pipeline (PGAP) (5), and curated using GenomeMatcher (6). While referring to annotation data obtained from the Microbial Genome Annotation Pipeline (http://www.migap.org/) (7), we corrected start codon positions and added genes that were missing in the PGAP annotation.

### Nucleotide sequence accession numbers.

The genome sequence of *Sphingomonas macrogoltabida* EY-1 has been deposited in GenBank under the accession numbers CP012700 to CP012705.
